# Arthropod Pest Control for UK Oilseed Rape – Comparing Insecticide Efficacies, Side Effects and Alternatives

**DOI:** 10.1371/journal.pone.0169475

**Published:** 2017-01-11

**Authors:** Han Zhang, Tom Breeze, Alison Bailey, David Garthwaite, Richard Harrington, Simon G. Potts

**Affiliations:** 1 Centre for Agri-Environmental Research, School of Agriculture, Policy and Development, University of Reading, Reading, Berkshire, United Kingdom; 2 Land Management and Systems, Faculty of Agribusiness and Commerce, Lincoln University, Christchurch, New Zealand; 3 Pesticide Usage Survey, Fera Science Ltd, Sand Hutton, York, United Kingdom; 4 Rothamsted Insect Survey, Rothamsted Research, Harpenden, United Kingdom; Chinese Academy of Agricultural Sciences Institute of Plant Protection, CHINA

## Abstract

Oilseed rape (*Brassica napus*) is an important combinable break crop in the UK, which is largely protected from arthropod pests by insecticidal chemicals. Despite ongoing debate regarding the use of neonicotinoids, the dominant seed treatment ingredients used for this crop, there is little publicly available data comparing the efficacy of insecticides in controlling key arthropod pests or comparing the impacts on non-target species and the wider environment. To provide an insight into these matters, a UK-wide expert survey targeting agronomists and entomologists was conducted from March to June 2015. Based on the opinions of 90 respondents, an average of 20% yield loss caused by the key arthropod pests was expected to have occurred in the absence of insecticide treatments. Relatively older chemical groups were perceived to have lower efficacy for target pests than newer ones, partly due to the development of insecticide resistance. Without neonicotinoid seed treatments, a lack of good control for cabbage stem flea beetle was perceived. Wide spectrum foliar insecticide sprays were perceived to have significantly greater negative impacts than seed treatments on users’ health, natural enemies, pollinators, soil and water, and many foliar active ingredients have had potential risks for non-target arthropod species in UK oilseed rape fields for the past 25 years. Overall, 72% of respondents opposed the neonicotinoid restriction, while 10% supported it. Opposition and support of the restriction were largely based on concerns for pollinators and the wider environment, highlighting the uncertainty over the side effects of neonicotinoid use. More people from the government and research institutes leaned towards neutrality over the issue, compared to those directly involved in growing the crop. Neonicotinoid restriction was expected to result in greater effort and expenditure on pest control and lower production (0–1 t/ha less). Alternatives for future oilseed rape protection were then discussed.

## Introduction

Oilseed rape (*Brassica napus*) production has increased in many European Union (EU) countries since 2005 when the EU set a target for 20% of its energy to be from renewable sources by 2020 [1]. In the UK, oilseed rape production reached 652,000 hectares in 2015 [2], increasing annually by an average 2% over the past ten years. UK oilseed rape is grown for vegetable oil, animal feed and biodiesel. It serves as a break crop with wheat production and accounts for about 80% of the UK combinable break crops [3,4].

Oilseed rape has many arthropod pest and disease problems, prompting widespread use of chemical insecticides [5]. Pesticide usage survey data collected by the UK’s Fera Science Ltd. ([6]; methods and figures see S1 Appendix) indicate that, between 1974 and 1988, pest control in oilseed rape in Great Britain was dominated by the use of organophosphate sprays and organochlorines as both foliar sprays and seed treatments. After 1990, pyrethroids quickly replaced these chemicals as foliar sprays, because of their higher efficacy in pest control and lower toxicity to humans, becoming the major insecticide group used from 1995 onwards. On average, pyrethroids represented 80% of total insecticidal weights, and 95% of total area treated for all foliar sprays used in oilseed rape in Great Britain for the past 25 years. Since the ban on organochlorines in 1999 (due to their high toxicity to users, and persistence and bioaccumulation in the environment), neonicotinoids have become the main seed treatments for oilseed rape in Great Britain, with 1,930 kg weight increase every two years. Three neonicotinoid active ingredients, clothianidin, thiacloprid and thiamethoxam, have been restricted in Europe since the end of 2013 [7], and the 2014/15 season was the first without these seed treatments being available.

The neonicotinoid restriction primarily resulted from the perceived negative lethal and sublethal impacts on both managed and wild pollinators [7]. Since the start of the restriction, the evidence base has been strengthened around the effects of neonicotinoids on crop production, human health, pollinators and the wider environment [8–11]. However, major gaps remain (e.g., concentration and toxicity of neonicotinoid metabolites in the environment, see [12]). Strongly contrasting views about the risks of using this chemical group in agriculture exist, and little consensus has been reached [10,11], with an ongoing argument in UK between the National Farmers Union (NFU) and government regarding the lifting of the restriction [13]. The neonicotinoid debate has resulted in uncertainties for the future of oilseed rape production and management for UK farmers, in terms of cropping areas, insecticide choices and impact on profits [14,15].

Two new chemical groups have recently been adopted by UK growers (azomethines and oxadiazines, from around 2012), which could potentially act as alternatives to neonicotinoids. However, as with all currently available chemical groups, there is little publicly available information concerning their relative efficacy in controlling key pests [8], or their side effects on non-target species and the wider environment. Thus it is difficult to assess the overall impact of using a specific insecticide chemical on oilseed rape.

In this study, based on experts’ opinions, we provide an insight into these matters by comparing the relative efficacies and side effects of insecticide chemical groups used in UK oilseed rape production. Experts’ opinions on the neonicotinoid restriction are also analysed. Because the survey finished in June 2015, the perceived inputs and yields are compared between 2013/14 and 2014/15 season. For future sustainable oilseed rape production in the UK, the potential alternative crop management options are thus discussed.

## Materials and Methods

### Survey structure

An online survey by Qualtrics [16] was conducted to collect UK experts’ opinions. The online survey has been approved by the ethics committee of the School of Agriculture, Policy and Development, University of Reading. As stated in the ethical approval (B165) and cover letter, the data can only be shared in summary forms, where no individual will be identified, and raw data will be destroyed in March 2018.

The survey had 28 questions (see S2 Appendix. Survey questionnaire) divided into four parts: (i) general section (6 questions including occupations, experience of giving farmer advice, and knowledge of crops), (ii) wheat section (7 questions, including yields, main pests and damage, insecticide efficacy, and importance of arthropod natural enemies), (iii) oilseed rape section (7 questions, as in the wheat section) and (iv) neonicotinoid section (8 questions, including opinions and reasons for the restriction, expected yields and inputs, and alternative pest management strategies). This paper refers only to the oilseed rape and neonicotinoid sections.

Respondents were also asked to give the regions they refer to when providing the information. To capture the degree of certainty, respondents were asked to give certainty scores on a 1–5 scale (5 being most certain) following several of the questions.

### Survey distribution

In January 2015, a pilot study using 15 experts, mainly from universities, was conducted. Because only minor changes to the wording of a small number of questions were made based on their feedback, these responses were included in the analyses. The main nationwide survey was carried out between March and June 2015. Potential respondents were contacted by searching through directories of universities, research institutes, NGOs (non-government organizations), government sectors, consulting firms, and agri-chemical companies. Universities were filtered via the Guardian league table 2015 by subjects of ‘Agriculture, forestry & food’ and ‘Biosciences’, and then relevant researchers were selected by scanning through the related department staff pages. Independent consultants were contacted through the Association of Independent Crop Consultants (AICC). Most experts were contacted by email and some through general enquiry web pages. Snowball sampling methods (where a respondent may pass the survey to other related experts) were also utilized to gain more respondents. A reminder with a link to the survey was sent to individuals two weeks after the initial request, and one more reminder two weeks later if no reply was received.

### Statistical analyses

#### Main arthropod pests and related damages in oilseed rape

Respondents were asked to rank the relative importance of the three most widespread arthropod pests (one being most important of the three) in oilseed rape in the past five years (2009/10 to 2014/15). These scales were used as weights (i.e., weights for the 1^st^, 2^nd^ and 3^rd^ most important pest were 0.5, 0.33 and 0.17 respectively, with a collective weight of 1) to produce a weighted average response for each pest. Answers were further categorised into different regions and the weighted average for each pest for each region was estimated. See S3 Appendix for the regional distribution.

Respondents were also asked to estimate, without insecticides, the direct feeding damage from each selected arthropod pest and the damage that Turnip yellows virus (TuYV; caused by *Myzus persicae*, peach—potato aphid) would have caused in the past five years, in terms of percentage yield loss (%), followed by the certainty scale (1–5, 5 being most certain). Average damage levels, standard deviations and certainty levels of the three most important pests were estimated.

#### Efficacy of insecticides and arthropod natural enemies

Information about the perceived efficacy of both chemical and natural pest control methods was requested in the survey.

The perceived efficacy of each available insecticide chemical group in the 2013/14 season was requested for each of the main pests selected from the previous question, following a 0–6 efficacy scale as below (Table 1):

**Table 1:** Insecticide efficacy scale.

0	1	2	3	4	5	6
Not sure	0% pest control	1–20% control	21–50% control	51–80% control	81–90% control	91–100% control

Pairwise efficacy comparisons among the available chemical groups were conducted by the Skillings–Mack [17,18] and related post hoc tests [19], using the software R 3.2.5 [20]. Responses were omitted if a respondent answered for only one chemical group for a specific pest. Due to the asymmetrical distribution and the non-parametric analyses for these ordinal data, medians and interquartile ranges were used to summarize the efficacy level.

The same efficacy and certainty scales were used to estimate the importance of arthropod natural enemies on pest control (without insecticides). Qualitative opinions on its importance for oilseed rape production were gathered by selecting from the following options: ‘Strongly agree’, `Agree’, `Neutral’,`Disagree’, `Strongly disagree’ and `Not sure’.

#### Side effects of insecticides on human health, environment and non-target arthropods

Perceived negative influences on users’ health, natural enemies, pollinators, water and soil were compared between the two application methods for UK oilseed rape(seed treatments and foliar sprays), using a 0–5 scale (0 being no influence, and 5 being greatest influence). Sign tests [21] were used to compare the perceived median differences between seed treatments and foliar sprays on these aspects. Responses were omitted if the respondent only answered for one of the two treatment types. Due to the asymmetrical distribution and the non-parametric analyses for these ordinal data, medians and interquartile ranges were used to summarize the side effect level.

Potential risks of common foliar active ingredients to the non-target arthropods for UK oilseed rape were also analysed by the in-field Hazard Quotient approach ([22]; see S4 Appendix for details). Hazard Quotient is the required assessment for pesticide registration in the European Union. The method combines the laboratory acute test [23] of two sensitive species (cereal aphid parasitoid *Aphidius rhopalosiphi* and predatory mite *Typhlodromus pyri*) and field application rate of the related foliar active ingredient [6] to produce a Hazard Quotient. If the quotient of either species exceeds two, the threshold level, it indicates a potential hazard of the active ingredient towards non-target arthropods. Due to the limited publicly available test results, only one representative active ingredient (the one that was used most often in UK oilseed rape and had test results available) was chosen from each available chemical group. For the same reason, and also that *Typhlodromus pyri* would normally be applicable in orchard fruit crops, only *Aphidius rhopalosiphi* was used as the test species.

#### Opinions on neonicotinoid seed treatment restrictions

A five-point Likert scale was used to measure expert opinions on the current neonicotinoid restrictions (‘Strongly favour’ to ‘Strongly oppose’ including a ‘Not sure’ option). Different possible reasons for holding favourable and unfavourable opinions were displayed for respondents to select. Fisher’s exact test was used (with Monte Carlo simulated p value, 100,000 replicates) [20] to explore whether or not experiences in farming services and the nature of the employing organization had an impact on respondents’ opinions.

## Results

### Response rates and information about respondents

In total, 455 online surveys were sent out, and 146 responses were received (43% effective response rate accounting for incorrect emails and declines), with 101 completed (70% of total responses). 90 people participated in the oilseed rape and neonicotinoid sections. The following analyses focused on these two sections.

From 2010 to 2014, 43% of respondents (39 out of 90) had worked for independent consultants/ consulting organizations (Fig E.1 in S5 Appendix). University staff accounted for 27%, government 17% and agri-chemical companies 16%. 65% of respondents had provided advice services to farmers.

### Main arthropod pests and related damage in oilseed rape (2009/10-2013/14)

**Fig 1:**
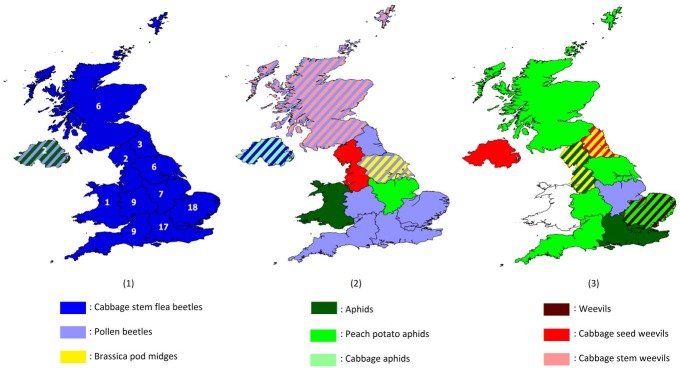
Perceived top three important arthropod pests of oilseed rape for each UK region. (1) Most important arthropod pests; (2) 2^nd^ most important arthropod pests; (3) 3^rd^ most important arthropod pests. Note that different regions are not directly comparable, due to different numbers of respondents for each region (see number in the first graph). The white area for Wales in the third graph denotes no data, since respondents only mentioned two pests. Stripes indicate two pests of equal importance. For the data see Table E.1 in S5 Appendix. Map shapefiles are from the GADM database of Global Administrative Areas and the Office for National Statistics [24,25], and software is QGIS [26].

Based on a weighted average of expert ranking, the three main arthropod pests in oilseed rape in the past five years were perceived to be cabbage stem flea beetle (*Psylliodes chrysocephalus*) (weighted average response = 28), pollen beetle (*Meligethes aeneus*) (18), and peach—potato aphid (*Myzus persicae*) (8). Different regions varied slightly (e.g., for the North West region, the second most important pest was cabbage seed weevil, and the equal third were aphids and brassica pod midge) (Fig 1; Table E.1 in S5 Appendix).

Due to the limited number of respondents, and the fact that some respondents represented several regions (up to four, S3 Appendix), Fig 1 represents a broad visualization. The following analyses were conducted without categorising into regions.

Without insecticide applications, the perception was that cabbage stem flea beetle would have caused the greatest direct damage, with a mean yield loss of 24% (certainty level 3). However, expert opinion on this also had the largest variation, with a standard deviation of 26%. Second was pollen beetle damage, with a mean value of 19%, and standard deviation of 20% (certainty level 3). Perceived peach—potato aphid direct damage averaged 13%, with a standard deviation of 10% (certainty level 3). TuYV loss without insecticides was perceived to be 15%, with a standard deviation 11%, and a certainty level of 2 (Table 2).

**Table 2:** The number of respondents, perceived mean crop yield loss (%) and certainty levels of the main arthropod pests (direct damage), and the turnip yellows virus in UK oilseed rape from 2009/10 to 2013/14 (without insecticides).

	Cabbage stem flea beetles	Pollen beetles	Peach potato aphids	TuYV
**Number of respondents**	56	41	19	66
**Mean damage (s.d.)**	24.2 (26.3)	18.9 (20.5)	13.2 (9.9)	15.0 (11.0)
**Mean certainty levels (s.d.)**	2.8 (1.1)	2.8 (1.0)	2.6 (1.3)	2.3 (1.2)

Note: Numbers in the brackets are the related standard deviations (s.d.). Certainty levels: 1–5, 5 being most certain.

### Efficacy of insecticides in oilseed rape (2013/14)

In terms of efficacy of the insecticide groups for oilseed rape protection, because of the potential complex annual variation in efficacy, only the available chemical groups used for the 2013/14 season were studied (Table 3).

**Table 3:** The perceived median insecticide efficacies (number of missing values; number of ‘Not sure’ responses; interquartile ranges) for the three main arthropod pests in UK oilseed rape in 2013/14.

Pests (number of respondents)	Carbamates	Pyrethroids	Neonicotinoid seed treatments	Neonicotinoid sprays	Oxadiazines	Azomethines
**Peach–potato aphid (16)**	2 (1; 1; 1–2) a	2 (0; 1; 1–3) a	5 (0; 0; 4–5.25) b	5 (2; 1; 5–6) bc	4 (6; 3; 4–4) c	4 (0; 1; 4–5.5) b
**Pollen beetle(26)**	1 (8; 4; 1–2.5) a	3 (2; 0; 2.75–4) a	1 (7; 3; 1–3) ab	4 (2; 2; 4–5) bd	4.5 (5; 8; 4–5) c	4 (5; 3; 3.5–5) d
**Cabbage stem flea beetle (44)**	1 (16; 4; 1–1.25) a	3 (1; 0; 3–3.5) b	5 (0; 0; 4–5) c	4 (8; 5; 2–5) c	3 (17; 13; 2–4) d	2 (17; 9; 1–3.75) d

Note: efficacy levels: 0 (Not sure), 1 (0% pest control), 2 (1–20%), 3 (21–50%), 4 (51–80%), 5 (81–90%), 6 (91–100%); ‘Not sure’ answers are omitted; different letters mean significant differences (α = 0.05) between two chemical groups (based on Skillings–Mack and related ad-hoc tests).

It should be noted that some chemical groups are not applied against some pests due to certain phenological and pharmacological factors. For example, neonicotinoid seed treatments are not normally used against pollen beetles, because they are primarily applied to autumn sown crops, whereas pollen beetles pose a threat in spring. Neonicotinoid sprays and oxadiazines are mainly used for this pest. Carbamates (mostly pirimicarb) are used mainly for aphid control, and azomethines are applied mainly against aphids and pollen beetles. This information was reflected in Table 3 by the perceived relatively low efficacy levels for the non-targeted pests.

Based on the pairwise comparisons among different chemical groups, neonicotinoids and the two newly introduced chemical groups (oxadiazines and azomethines) were perceived to have higher efficacy than carbamates and pyrethroids for peach–potato aphid (81–90% and 1–20% respectively) and pollen beetle control (51–80% and 21–50% respectively). Neonicotinoid seed treatments were perceived to have more efficacy than pyrethroids against cabbage stem flea beetle (81–90% and 21–50% respectively).

### Side effects of insecticides in oilseed rape

Comparing the two main insecticide application methods for UK oilseed rape, foliar sprays were thought to have significantly more negative impacts than seed treatments on users’ health, natural enemies, pollinators, water and soil (Table 4). The median influence levels (from 0, no influence, to 5, greatest influence) across all categories were perceived to be 3 for sprays and 1 for seed treatments.

**Table 4:** Number of pairs, medians (interquartile ranges) of the influence scale (0–5) of the general seed treatments and foliar sprays, and the sign tests of the perceived median differences in side effects between seed treatments and foliar sprays.

Categories	Number of pairs	Seed treatments	Sprays	Sign tests (α = 0.05)
**Health**	63	1 (0–1)	2 (1.5–3)	<0.00001
**Natural enemies**	64	1 (1–2)	4 (3–5)	<0.00001
**Pollinators**	61	1 (1–2)	4 (3–4)	<0.00001
**Water**	59	1 (1–2)	3 (2–4)	<0.00001
**Soil**	55	1 (1–2)	2 (1–3)	0.02

Note: influence scale: 0–5, 0 being no influence, 5 being greatest influence.

**Fig 2:**
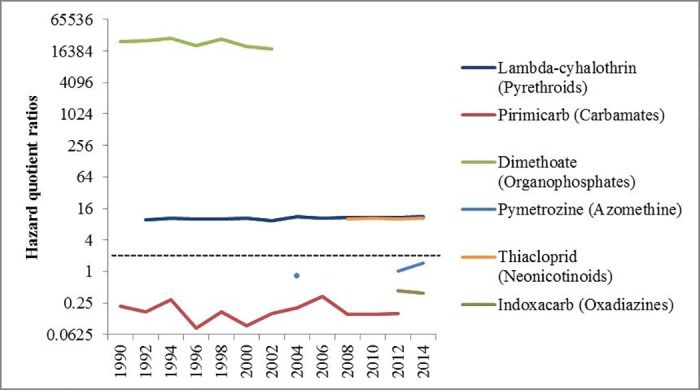
Hazard Quotient ratios of insecticide active ingredients used in oilseed rape in Great Britain (1990–2014). The test species is the aphid parasitoid *Aphidius rhopalosiphi*. Trigger value is 2 (marked with the black dashed horizontal line), above which indicates potential hazard of an active ingredient on non-target arthropod species. Y axis is the log scaled with base 4.

Focusing on the Hazard Quotients for foliar sprays, different active ingredients have large differences in terms of the potential risks to non-target arthropods. Dimethoate has much higher potential hazard levels, with lambda-cyhalothrin and thiacloprid following. In comparison, pymetrozine, indoxacarb and pirimicarb pose lower potential risks towards non-target arthropods (Fig 2).

### Uncertain future–neonicotinoid debates

From the information gathered, 72% (65 of 90) participants either opposed or strongly opposed the neonicotinoid restrictions in the UK while only 10% (9 of 90) supported or strongly supported this policy. 13 respondents took a neutral stance, and three respondents were not sure about this proposition (Fig E.2 in S5 Appendix).

**Fig 3:**
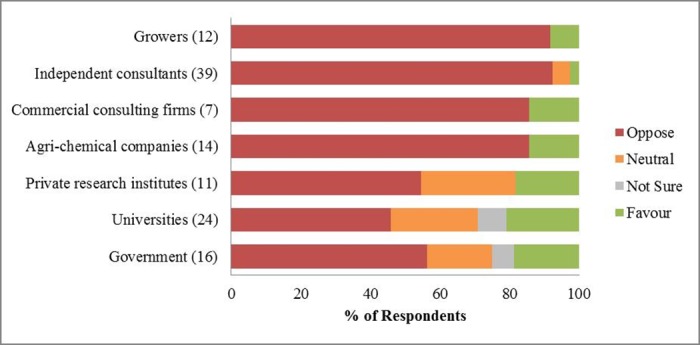
Percentage of respondents’ opinions on neonicotinoid restriction by organization type. *Oppose* includes ‘Strongly oppose’ and ‘Oppose’ options, *favour* includes ‘Strongly favour’ and ‘Favour’ options. Numbers in the brackets are the number of respondents from that organization type who answered the question.

In terms of organizations (NGOs and food industries were omitted, due to low respondent numbers), an average of 51% respondents from the government, universities, and private research institutes (Group A) opposed the neonicotinoid restriction, compared with 90% of respondents from the agri-chemical companies, commercial/independent consultants, and growers (Group B) (Fig 3). This difference was driven less by respondents who supported the restriction (an average of 20% from Group A compared with 7% from Group B), but more by those who held ‘Neutral’ and ‘Not sure’ opinions (30% compared with 3%). To compare this more quantitatively, we regrouped the six options in two ways: one was *oppose* (including ‘Oppose’ and ‘Strongly oppose’) versus *favour* (including ‘Favour’ and ‘Strongly favour’); the other was having an opinion (*oppose*/ *favour*) versus ‘Neutral’/ ‘Not sure’. Fisher’s exact test was used to test differences among organizations regarding their opinions on this issue. Results showed that there was no clear difference among organizations for whether to favour or oppose neonicotinoid restriction, but more people from Group A than Group B had a neutral/ not sure proposition (Table E.2 in S5 Appendix).

**Fig 4:**
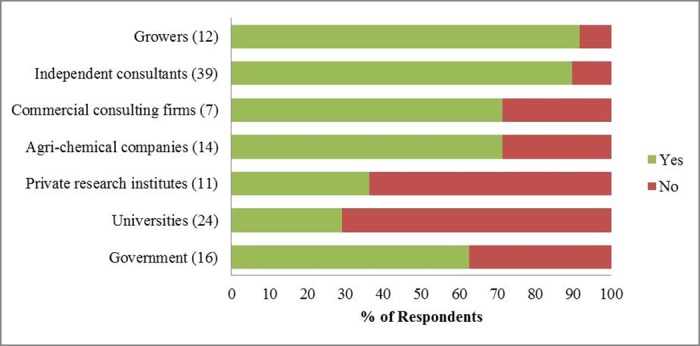
Percentages of respondents who have provided advice services to farmers by organization type (2009/10–2013/14). Numbers in brackets are the number of respondents from that organization type who answered the question.

Fisher’s exact test also showed a clear division between Group A and B regarding whether a respondent had provided advice services to farmers in the past five years (Table E.3 in S5 Appendix). An average of 41% from Group A and 85% from Group B had done so (Fig 4).

**Fig 5:**
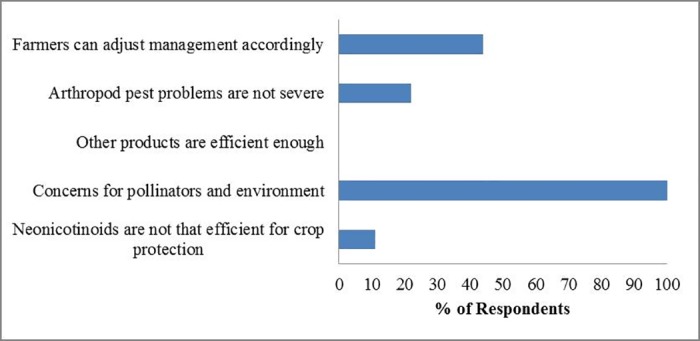
Reasons for favouring neonicotinoid restriction. Total number of respondents is 9.

**Fig 6:**
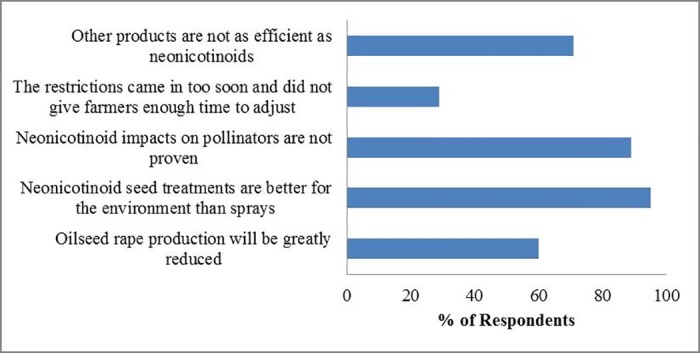
Reasons for opposing neonicotinoid restriction. Total number of respondents is 65.

Although only 10% (9) of respondents favoured the neonicotinoid restriction in the UK, all of them expressed concerns about the negative impacts neonicotinoids could have on pollinators and the wider environment. Four respondents believed that farmers can adjust to the management accordingly, and two suggested that the arthropod pest problems were not severe (Fig 5). Of the 65 respondents that opposed the restriction, the two most widespread reasons were also around environment and pollinators (95% and 89% respectively). 71% of respondents worried that other products are not as efficient as neonicotinoids, and 60% thought that oilseed rape production will be greatly reduced (Fig 6).

### 2014/15 VS 2013/14 oilseed rape growing season

**Fig 7:**
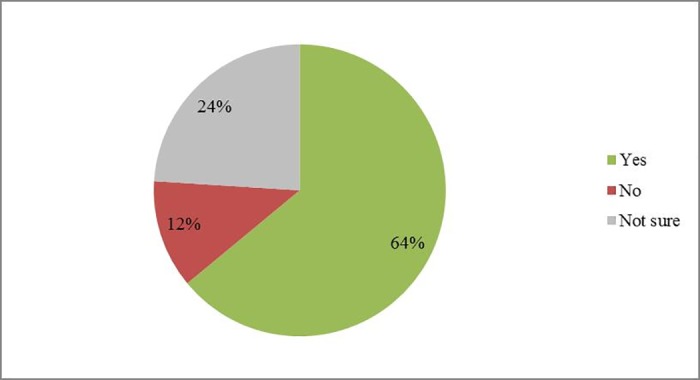
Whether more time was spent by agronomists inspecting oilseed rape fields for arthropod pest abundance. This is a 2014/15 compared with 2013/14 season. Total number of respondents is 75.

**Fig 8:**
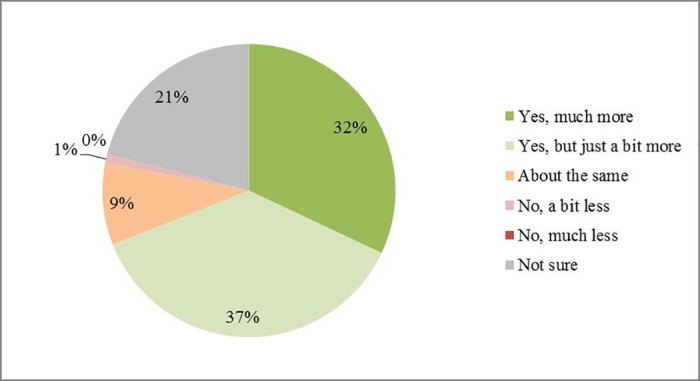
Percentage of respondents regarding whether they thought more money was spent on insecticide products. This is a 2014/15 compared with 2013/14 season. Total number of respondents is 78.

**Fig 9:**
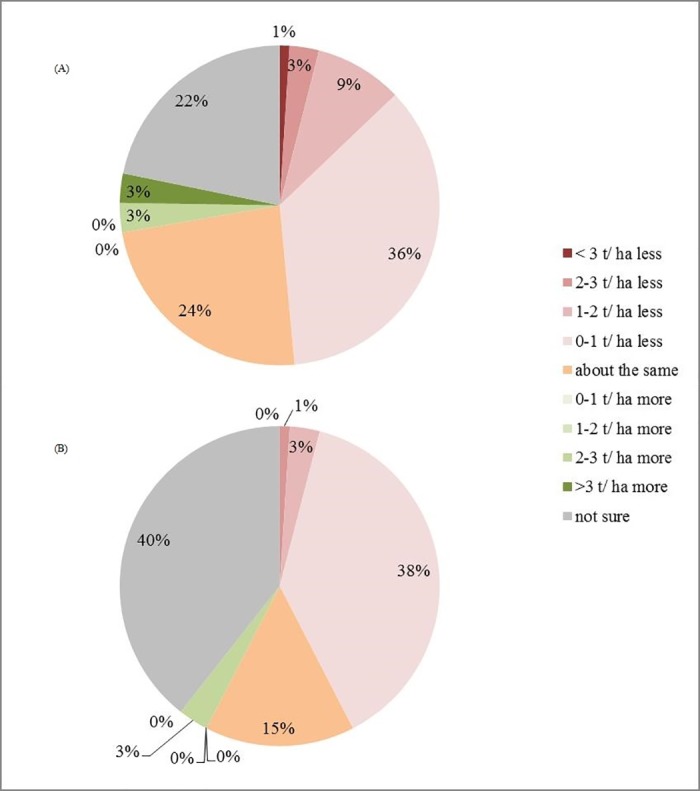
**(A) Expected winter and (B) spring oilseed rape yield in 2014/15 compared with 2013/14.** Total number of respondents is 76 and 71 respectively.

For the 2014/15 season (compared with 2013/14), 64% of respondents (48 out of 75) felt that more time had been spent by agronomists inspecting crops since the restrictions (Fig 7). 70% of respondents (54 out of 78) indicated that oilseed rape farmers spent more money on insecticide products during the 2014/15 season, but 37% (29 out of 78) thought that just a bit more had been spent (Fig 8). Only one respondent thought that farmers spent a bit less than 2013/14, while 15 were unsure. Most of the 76 respondents expected that the winter oilseed rape harvest in 2014/15 season would be between 0–1 t/ha less or about the same as the previous year (36% and 24% respectively). 22% of respondents were not sure about the answer, which was to be expected since yield changes are influenced by many factors in addition to pesticide use. Similar patterns occurred regarding the spring oilseed rape harvest. As the survey finished in June, yields for the year 2014/15 were estimated by the respondents directly (Fig 9).

### Alternative pest control options

**Fig 10:**
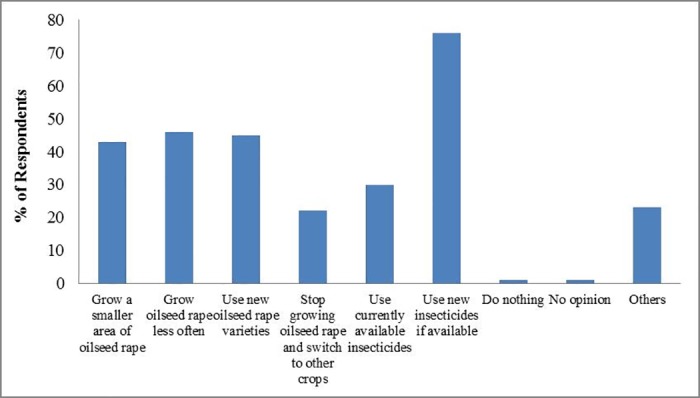
Perceived future management options if neonicotinoid seed treatments were withdrawn completely. Total number of respondents is 87.

**Fig 11:**
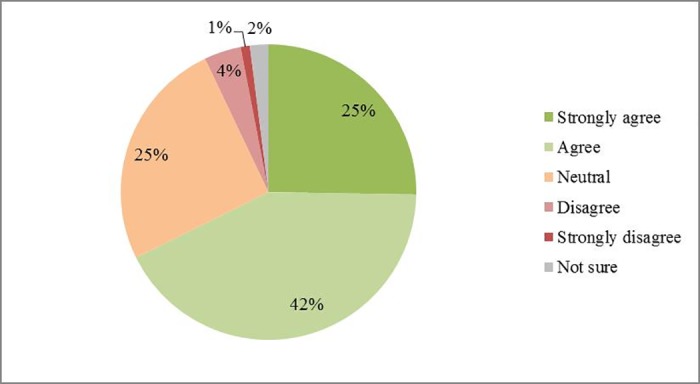
Perceived importance of arthropod natural enemies to oilseed rape production in UK. Total number of respondents is 83.

**Fig 12:**
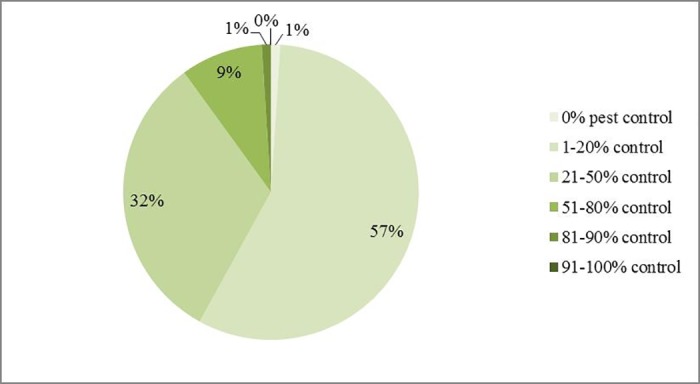
Without insecticides, perceived efficacy of arthropod natural enemies to control key pests in oilseed rape. Total number of respondents is 81.

In a hypothetical situation where neonicotinoids would be permanently banned in the UK, respondents’ suggestions towards alternative pest management strategies differed: 76% respondents (66 out of 87) would choose to use new insecticides if available. Other options such as ‘Grow oilseed rape less often’, ‘Use new oilseed rape varieties’, and ‘Grow a smaller area of oilseed rape’ were similar in terms of support (46%, 45% and 43% respectively). The use of currently available insecticides was mentioned by 30% of respondents. For the ‘Others’ option, 65% (13 out of 20) respondents mentioned the use of biocontrol/ IPM (integrated pest management) as an alternative approach (Fig 10). Focusing on natural pest control, 67% (56 out of 83) of respondents agreed that natural enemies are important for oilseed rape production (Fig 11). Without insecticide treatments, 57% of respondents thought that natural enemies could exert 1–20% control on oilseed rape pests, and 32% suggested 21–50% control (Fig 12).

## Discussion

### Main arthropod pests and related damage in oilseed rape (2009/10-2013/14)

The most important arthropod pests in UK oilseed rape based on perceptions from the survey (cabbage stem flea beetle, pollen beetle, and peach—potato aphid) were also indicated by [27] on a European scale, except for the peach—potato aphid, which was considered a minor pest in Europe. However, peach—potato aphid is an important pest in the UK, especially as a vector of TuYV ([28] showed an average 15% yield loss by this virus in untreated crops).

The large variation in levels of perceived pest damage suggests that pest damage is likely to vary greatly under different contexts. [29] suggested a 1% untreated yield loss for cabbage stem flea beetle, 0.5% for pollen beetle, and 3% for aphids carrying TuYV. In 2010, [30] found varied pollen beetle damage on different study sites in England (0–6% yield loss). The low certainty levels provided by experts (an average of 3 out of 5) suggest that uncertainties and research gaps remain in this area.

### Efficacy of insecticides in oilseed rape (2013/14)

Due to the commercial confidentiality and expensive trials [8], it is difficult to obtain data on insecticide efficacy which can differ among active ingredients within one chemical group [31]. Efficacy levels are also difficult to evaluate and compare because many factors can affect them both temporally and spatially, including insecticide resistance (Table E.4 in S5 Appendix), application methods, plant growth stages, etc. [32,33].

The perceived high efficacy of neonicotinoids against insect pests and related diseases has been demonstrated in several studies ([34]: cabbage stem flea beetle; [35]: pollen beetle; [36]: peach—potato aphid), although others have shown limited efficacy ([37]: peach—potato aphid). For the relatively new groups, oxadiazines and azomethines, the perceived relatively high efficacy against pollen beetle was illustrated in a recent German study [38]. However, limited efficacy by these two groups was also found [39,40]. It should be noted that without careful management (e.g., rotating insecticides with different available modes of action, [41,42]), resistance to the above insecticides could occur for pests in UK oilseed rape, thus reducing the efficacy. Neonicotinoid resistance has already been detected in the peach—potato aphid in Southern Europe [43].

Pyrethroids and carbamates had the lowest expected efficacy against the peach—potato aphid (1–20%), which could be partly due to the insecticidal resistance occurred for this pest in UK (Table E.4). Although in 2010, a HGCA study found that pyrethroids were efficient against pollen beetle (about 90% control, [30]), experts in our study only expressed a median 21–50% control, possibly due to growing resistance since its appearance in 2006 (Table E.4 in S5 Appendix).

The perceived low confidence in cabbage stem flea beetle control is worrying. Apart from neonicotinoid seed treatments, the other available chemical pyrethroids were perceived to exert lower than 50% control, which could partly due to the resistance occurred in this pest (Table E.4 in S5 Appendix) [44,45].

Across all experts, the perceived efficacy rarely exceeded 90% from all available chemical groups used in UK oilseed rape in 2013/14. This may reflect the lack of confidence in assigning efficacy for insecticides used in oilseed rape, but also the fact that many insecticides, even when newly introduced, cannot provide 90% pest control [46]. The perceived low and/ or uncertain efficacy in the available chemical groups and the uncertainty in pest control after the neonicotinoid restriction indicate an urgent need for robust, accountable and updated information about efficiency of insecticides for pest control for this crop in the local fields.

### Side effects of seed treatments versus sprays

Experts generally perceived seed treatments to be less harmful than sprays. This is to be expected as: (i) compared with sprays, seed treatments have less direct contact with operators and non-target species [36]; (ii) less surface runoff [47]; and (iii) reduced concentration in the environment [48]. However, many counter-arguments have arisen in the past few years against this application method, especially for the neonicotinoid group. Traces of neonicotinoid residues have been detected in humans [49], pollinators and beehives [50,51], soil [52] and water [53]. Negative impacts have also been found on human health [54], pollinators [8,55], natural enemies [10], earthworms [56] and aquatic invertebrates [53]. Possible reasons behind these side effects are the systemic characteristics of neonicotinoids (residues in treated plant tissues) [12], low soil absorption (high leaching potentials) [57], and great toxicity to invertebrates [53].

On the other hand, sprays can also cause side effects for human health and the wider environment. For example, although relatively few human poisonings from pyrethroids have been reported despite their extensive use worldwide [58], sub-lethal reactions have been found, including paresthesia and nausea [58]. Negative impacts have also been detected for pollinators [50,59–61], natural enemies [62–64], soil [65–68] and water systems [69,70].

Since the survey finished in June 2015, more knowledge has been accumulated on the side effects of insecticides toward non-target species and the wider environment. Take the impacts of neonicotinoid seed treatments on pollinators as an example: since the research gaps were identified by [71] (a literature review up to June 2015), many studies have investigated other active ingredients besides imidacloprid [72], pollinator species besides honey bees (*Apis*) [73], and/or impacts on the colony besides individual development [74]. Although more work needs to be done to improve the evidence base on this issue [75], respondents’ opinions could be changed by the new evidence.

However, with the currently available evidence, it is still difficult to compare the overall side effects of the two insecticidal application methods, especially between neonicotinoids and pyrethroids, since little research has done so (but see [76] on earthworms).

### Hazard Quotient ratios from sprays

By using the in-field Hazard Quotient method, a temporal comparison of hazard levels on non-target arthropods among available foliar active ingredients for UK oilseed rape was presented (Fig 2). However, limitations exist in interpreting the results because this method is based on the laboratory acute toxicity tests, one being that these tests are difficult to account for the influence from the environment (S4 Appendix) [77,78].

The high toxicity of dimethoate to human health and the wider environment has been widely recognized [79]. Although its use in UK oilseed rape was stopped a decade ago, it is still approved for use in wheat. The high threat potential of lambda-cyhalothrin towards non-target arthropods has also been reflected in previous studies [80–82]. Although thiacloprid has been shown to have negative effects on arthropod natural enemies [40], it has been found to have limited side effects on bees by some studies [36,83]. According to [84], pymetrozine has less of an effect than pirimicarb on aphid natural enemies. Indoxacarb has been shown to be less toxic than lambda-cyhalothrin to arthropod predators and parasitoids [85,86], but its potential hazard to honey bees could be high [87]. Relatively low side effects from pirimicarb against natural enemies have also been recorded [88].

### Perceptions on neonicotinoid restriction

The tendency for respondents from the universities, private research institutes and government to choose the ‘Neutral’ opinion on the neonicotinoid restriction debate is worth discussing. These respondents may have had an actual mid-point opinion on this issue, that they neither opposed nor favoured the restriction. It may have been because there was a lack of interest in this topic, or they considered similar overall costs and benefits for either side [89].

Uncertainty could also have been important to this group: they may have had more recent information about the effects of neonicotinoids on crop yields, pollinators and the wider environment, but because of the complexity of this issue and gaps in the current evidence base [8], they could not estimate the net costs and benefits of neonicotinoids. This is also reflected in that the reasons most frequently chosen for both the *oppose* and *favour* groups were all around pollinators and the wider environment (Figs 5 and 6). On the other hand, they may have been less well informed about the field situations or other related risks for farmers than consultants [90], who have provided more advice services to farmers (Fig 4, Table E.3 in S5 Appendix).

It is also possible that some respondents chose the neutral option to avoid the cognitive costs of selecting the most appropriate opinion, even though they may lean towards one side [91], while others chose this midpoint as a ‘hidden don’t know’ [92]. Nevertheless, the proportion of the last reason is estimated to be small in this study, since a ‘Not sure’ was included as an option to avoid the ambiguity [93], and online surveys potentially have less of this issue since people are more free to express their true opinions [94].

### Expected time, money and yield comparisons between 2013/14 and 2014/15

To our knowledge, little publicly available information has been made available on the time and money spent before and after the neonicotinoid restrictions, and limited research has been done regarding the impact of neonicotinoids on crop yield [8], but see [15,95,96]. According to the average response on this issue, compared with 2013/14 season, agronomists spent more time on inspecting oilseed rape crops for pest damage, farmers spent more money for insecticide purchases, and crop yield would be reduced in 2014/15.

Changes in the time spent on pest control activities should be taken into account when considering the pros and cons of neonicotinoid seed treatments, because it represents a hidden benefit if agronomists/ farmers spend less time on pest control, but more time on other activities. As for insecticide purchases, [15] indicates a negative relationships between neonicotinoid seed treatments and foliar sprays for UK oilseed rape, which could potentially lead to more insecticide costs to farmers after the restriction [28].

Although most respondents expected lower oilseed rape yield in 2014/2015 than 2013/2014, this does not reflect the actual average yields (3.9 and 3.6 t/ ha respectively, [2]). It is difficult to assess the impact of neonicotinoid restriction on the yield change based on one year data, since the increased yield may well be due to nicer weather and lower pest pressure during the year [2].

### Alternative methods of pest control in oilseed rape

The results from this study suggest a clear preference towards using new insecticides if these become available. This partly reflected a lack of confidence in the old chemical groups, but also an acknowledgment of the importance of insecticides for crop protection [97]. Developing new insecticides (especially with new modes of action) would help current insecticide resistance problems in oilseed rape. However, insecticide discovery has been a challenge, with the shrinking number of agri-chemical companies involved in the research, and the expensive and time-consuming development process [98].

Respondents also advised growers to use new oilseed rape varieties if available. Indeed, crop breeding in the UK has contributed to yield protection by improving crop resistance to pests and diseases [99]. A new oilseed rape variety ‘Amalie’ has been recommended for use against TuYV in the 2016/17 growing season [100], and a recently completed pre-breeding project has further explored the potential to develop commercial oilseed rape varieties to tolerate this virus infection [101].

With those who advised farmers to grow small areas of oilseed rape in the future, this concern has also been expressed through a farmer survey during 2014/15 by the Farm Business Survey (FBS) team [14], where the most important reasons for a future reduction in area were crop rotations, reduced crop price, and cabbage stem flea beetle damage. When comparing the 2014/15 with 2013/14 [2], the total area has decreased by about 3% (22,000 ha).

In line with the experts’ suggestions, the importance of IPM has also been emphasized by EU and UK policymakers [102], and numerous UK organizations (e.g., LEAF -Linking Environment and Farming, Natural England). In order to develop further IPM for oilseed rape production, one of the most crucial aspects is to understand insecticide efficacy on pest control, and its changes over time due to resistance: this will be important for developing action thresholds to use chemicals strategically [32]. Another crucial aspect of IPM, as expressed by experts in this study, is natural pest control. Many studies have been carried out to evaluate the impact of natural enemies on pest suppression in oilseed rape, and to seek methods of conserving them [27]. However, knowledge gaps still exist in this area [27].

A big challenge will be to combine these two aspects when developing IPM strategies, so that the side effects of insecticides on natural enemies could be reduced to a minimum. Indeed, by conducting research among eight EU countries, [103] have found consistent negative effects of insecticides on biological control potential.

Farmers would adopt new strategies only if they work better than current practices [102]. Profit is one fundamental aspect in the judgement. However, to our knowledge, little literature is available which estimates the influence of natural enemies on crop yield or net profits, especially for large scale field crops by conventional or IPM farmers [104,105], and none has focused on oilseed rape, partly because of the difficulty of conducting field experiments. More research is needed to estimate the economic value of this important service provided by natural enemies.

## Conclusion

Insecticides used in UK oilseed rape production have been designed to be more efficient in controlling pests, and less harmful to non-target species and the wider environment. However, their efficacy levels are not fully understood, and may not be sufficient in the long-term, due to the limited publicly available studies and fast development of insecticide resistance in pest species. Similarly, it is difficult to assess their side effects, partly because little research has comprehensively compared the impacts of different insecticides in a standardised manner. The type and extent of benefits for farmers are also fundamental when assessing insecticides. For these reasons, the decision as to whether further to restrict neonicotinoid seed treatments in oilseed rape needs careful evaluation. It is a challenge to take into account the multi-faceted aspects when assessing an insecticide; one way forward could be to translate each aspect into economic values, and then apply cost benefit analysis. In order to do so, more research is needed regarding the influence of a chemical on crop protection, farm profit, the environment and related ecosystem services. This study provided an insight into these aspects, but limitations exist due to a relatively small sample of expert opinions.

Integrated pest management presents an important potential future strategy for oilseed rape production, and the importance of insecticides and natural pest control should be better recognised and incorporated. Economic valuation of pest control services by natural enemies for oilseed rape needs to be quantified, coupled with improved communication and knowledge exchange between government, researchers, consultants and growers.

## Supporting Information

S1 Appendix Insecticide development for oilseed rape protection in UK: methods and figures. (DOCX)

S2 Appendix Questionnaire. (DOCX)

S3 Appendix Regional distributions. (DOCX)

S4 Appendix Hazard Quotient approach. (DOCX)

S5 Appendix Other figures and tables. (DOCX)
