# Supplementary material for: Arthropod Pest Control for UK Oilseed Rape – Comparing Insecticide Efficacies, Side Effects and Alternatives
Source: PLoS One. 2017 Jan 11;12(1):e0169475. doi: 10.1371/journal.pone.0169475 (PMC5226783; doi:10.1371/journal.pone.0169475)
Supplement: S1 Appendix — (DOCX) [file pone.0169475.s001.docx]

**S1 Appendix. Insecticide development for oilseed rape protection in UK: methods and figures**

Information on insecticides was collected from the Fera pesticide usage surveys [1] . Since 1990, the surveys on oilseed rape have been conducted biennially, and did not include Northern Ireland until 2010. However, oilseed rape has not been widely grown in Northern Ireland. In order to be comparable, information for Northern Ireland was omitted. Foliar sprays and seed treatments were analysed separately by insecticide chemical groups, and total weights (kg), total area treated (ha) and application rates (g/ha) were compared.

Before 1990, only four surveys of arable crops, including oilseed rape, were conducted, mainly targeting England and Wales. Due to the lack of information on the formulated mixtures within individual products, the total weight of individual active ingredients cannot be separated. Thus only the total treated areas (ha) with individual chemical groups could be compared.

Fig. A.1. Total treated area (ha) for various chemical groups in England and Wales pre-1990 (excluding seed treatments).

Fig. A.2. Total treated areas (ha) for various chemical groups in England and Wales pre-1990 (Seed treatments only).

Fig. A.3. Total weights (kg) for various chemical groups in Great Britain post- 1990 (excluding seed treatments).

Fig. A.4. Total treated areas (ha) for various chemical groups in Great Britain post- 1990 (excluding seed treatments).

Fig. A.5. Application rates (g/ha) for various chemical groups in Great Britain post- 1990 (excluding seed treatments).

Fig. A.6. Total weights (kg) for various chemical groups in Great Britain post- 1990 (seed treatments only).

Fig. A.7. Total treated areas (ha) for various chemical groups in Great Britain post- 1990 (seed treatments only).

Fig. A.8. Application rates (g/ha) for various chemical groups in Great Britain post- 1990 (seed treatments only).

**References**

1. Fera. Pesticide Usage Surveys; 2015. Fera Science Ltd [Internet]. Accessed: http://pusstats.fera.defra.gov.uk/index.cfm.
