# Supplementary material for: Arthropod Pest Control for UK Oilseed Rape – Comparing Insecticide Efficacies, Side Effects and Alternatives
Source: PLoS One. 2017 Jan 11;12(1):e0169475. doi: 10.1371/journal.pone.0169475 (PMC5226783; doi:10.1371/journal.pone.0169475)
Supplement: S2 Appendix — (DOCX) [file pone.0169475.s002.docx]

**S2 Appendix. Questionnaire**

Section 1: Some questions about you

* Could you create a code of 4-6 letters/ numbers, in case if you'd like to withdraw from the study later?

1. What type(s) of organizations have you worked for in the last 5 years? Tick all that apply.

- Government
- Universities
- Private research institutes
- Agri-chemical companies
- NGOs
- Commercial consulting firms
- Independent consultants/ consulting organizations
- Food industries
- Growers
- Other, please state: ____________________

2. Do you provide or have you provided advice services to farmers in the last 5 years?

- Yes
- No

3. Are you familiar with some aspects about arthropod pests (e.g: aphids), and related control methods (insecticides and natural enemies) in wheat?

- Yes
- No

(Note: For online version, if answered for No, the survey would be terminated, and answers would be recorded. The same applied for Question 4.)

4. Are you familiar with some aspects about arthropod pests (e.g: aphids), and related control methods (insecticides and natural enemies) in oilseed rape?

- Yes
- No

Section 2: Wheat crop, pests, and related control methods (2009/10-2013/14)

5. Which county/ region/ country(s) in UK do you refer to when providing wheat information for this section?

6. What have been the typical wheat yields in the last 5 years?

|  | Yield (t/ha) |
| --- | --- |
| Winter wheat |  |
| Spring wheat |  |

7. Please rank from 1-3 (1 being most important) the 3 most significant pests for wheat (2009/10-2013/14). If you are not sure about the specific species (e.g.: Rose—grain aphids), just choose the general term (e.g.: Aphids).

______ Aphids

______ Rose—grain aphids

______ Grain aphids

______ Bird cherry—oat aphids

______ Flies

______ Gout fly larvae

______ Wheat bulb fly larvae

______ Yellow cereal fly larvae

______ Grey field slugs

______ Wireworms

______ Leatherjackets

______ Orange wheat blossom midges

______ Other 1

______ Other 2

______ Other 3

8. Without insecticide treatments, how much wheat yield loss (%) would have been typically caused by direct feeding in the last 5 years (could be a range or a number)? Referring to the pests from Question 7. Please also provide how certain you are about each answer (1-5, 5 being most certain).

|  | Wheat yield losses (%) | Certainty scale (1-5) |
| --- | --- | --- |
| 1^st^ important pest |  |  |
| 2^nd^ important pest |  |  |
| 3^rd^ important pest |  |  |

(Note: for the online version, answers provided in Question 7 would be automatically copied to the first column of Question 8 and 9.)

9 . How efficient do you think the following insecticide chemical groups used in wheat protection were in 2013/14?  Referring to the pests from Question 7.

Please use the 0-6 efficacy labels as below:

| 0 | 1 | 2 | 3 | 4 | 5 | 6 |
| --- | --- | --- | --- | --- | --- | --- |
| Not sure | 0% pest control | 1-20% control | 21-50% control | 51-80% control | 81-90% control | 91-100% control |

|  | Carbamates (eg: Aphox) | Organophosphates (eg: Govern) | Pyrethroids (eg: Alert) | Neonicotinoid seed treatments (eg: Deter) | Neonicotinoid sprays (eg: Biscaya) | Flonicamids (eg: Teppeki) | Others (please specify below with efficacy after) |
| --- | --- | --- | --- | --- | --- | --- | --- |
| 1^st^ important pest |  |  |  |  |  |  |  |
| 2^nd^ important pest |  |  |  |  |  |  |  |
| 3^rd^ important pest |  |  |  |  |  |  |  |

10. Without insecticide treatments, how much yield loss would have been typically caused by the Barley yellow dwarf virus in the last 5 years (could be a range or a number)?         Please also provide how certain you are about this answer (1-5, 5 being most certain).

|  | Wheat yield losses (%) | Certainty scale (1-5) |
| --- | --- | --- |
| Barley yellow dwarf virus |  |  |

11. To what extent do you agree or disagree that, arthropod natural enemies (ladybirds, ground beetles, spiders, etc.) are important for wheat production?

- Strongly Agree
- Agree
- Neutral
- Disagree
- Strongly Disagree
- Not sure

12. Without insecticides, how much do you think arthropod natural enemies as a whole could control key pests in wheat?

- 0% pest control
- 1-20% control
- 21-50% control
- 51-80% control
- 81-90% control
- 91-100% control

    * How certain are you about this question (1-5, 5 being most certain)?

Section 3: Oilseed rape crop, pests, and related control methods (2009/10-2013/14)

13. Which county/ region/ country(s) in UK do you refer to when providing oilseed rape information for this section?

14. What have been the typical oilseed rape yields in the last 5 years?

|  | Yield (t/ha) |
| --- | --- |
| Winter oilseed rape |  |
| Spring oilseed rape |  |

15. Please rank from 1-3 (1 being most important) the 3 most significant pests for oilseed rape in the last 5 years. If you are not sure about the specific species (e.g.: Peach—potato aphids), just choose the general term (e.g.: Aphids).

______ Aphids

______ Peach—potato aphids

______ Cabbage aphids

______ Brassica pod midges

______ Beetles

______ Pollen beetles

______ Cabbage stem flea beetles

______ Weevils

______ Cabbage seed weevils

______ Cabbage stem weevils

______ Other 1

______ Other 2

______ Other 3

16. Without insecticide treatments, how much oilseed rape yield loss (%) could have been typically caused by direct feeding in the last 5 years (could be a range or a number)? Referring to the pests from Question 15.  Please also provide how certain you are about each answer (1-5, 5 being most certain).

|  | Wheat yield losses (%) | Certainty scale (1-5) |
| --- | --- | --- |
| 1^st^ important pest |  |  |
| 2^nd^ important pest |  |  |
| 3^rd^ important pest |  |  |

(Note: for the online version, answers provided in Question 15 would be automatically copied to the first column of Question 16 and 17.)

17. How efficient do you think the following insecticide chemical groups used in oilseed rape protection were in 2013/14 season? Referring to the pests from Question 15. Please use the 0-6 efficacy labels as below:

| 0 | 1 | 2 | 3 | 4 | 5 | 6 |
| --- | --- | --- | --- | --- | --- | --- |
| Not sure | 0% pest control | 1-20% control | 21-50% control | 51-80% control | 81-90% control | 91-100% control |

|  | Carbamates (eg: Aphox) | Pyrethroids (eg: Alert) | Neonicotinoid seed treatments (eg: Chinook) | Neonicotinoid sprays (eg: Biscaya) | Oxadiazines (eg: Explicit) | Azomethines (eg: Plenum) | Others (please specify below with efficacy after) |
| --- | --- | --- | --- | --- | --- | --- | --- |
| 1^st^ important pest |  |  |  |  |  |  |  |
| 2^nd^ important pest |  |  |  |  |  |  |  |
| 3^rd^ important pest |  |  |  |  |  |  |  |

18. Without insecticide treatments, how much yield loss would you estimate to have been typically caused by the Turnip yellows virus in the last 5 years (could be a range or a number)?

Please also provide how certain you are about this answer (1-5, 5 being most certain).

|  | Oilseed rape yield losses (%) | Certainty scale (1-5) |
| --- | --- | --- |
| Turnip yellows virus |  |  |

19. To what extent do you agree or disagree that arthropod natural enemies (ladybirds, ground beetles, spiders, etc.) are important for oilseed rape production?

- Strongly agree
- Agree
- Neutral
- Disagree
- Strongly disagree
- Not sure

20. Without insecticides, how much do you think arthropod natural enemies as a whole could control key pests in oilseed rape?

- 0% pest control
- 1-20% control
- 21-50% control
- 51-80% control
- 81-90% control
- 91-100% control

    * How certain are you about this question (1-5, 5 being most certain)?

Section 4: Neonicotinoid seed treatment restriction in oilseed rape

21. Which county/ region/ country(s) in UK do you refer to with this neonicotinoid section?

22. To what extent do you favour or oppose the restriction on the neonicotinoid seed treatments in oilseed rape?

- Strongly favour
- Favour
- Neutral
- Oppose
- Strongly oppose
- Not sure

      * Why do you pick ‘Favour’ or ‘Strongly favour’? Tick all that apply.

- Neonicotinoids are not that efficient for crop protection
- Concerns for pollinators and environment
- Other products are efficient enough
- Arthropod pest problems are not severe
- Farmers can adjust management accordingly
- Others ____________________

   * Why do you pick ‘Oppose’ or ‘Strongly oppose’? Tick all that apply.

- Oilseed rape production will be greatly reduced
- Neonicotinoid seed treatments are better for the environment than sprays
- Neonicotinoid impacts on pollinators are not proven
- The restrictions came in too soon and did not give farmers enough time to adjust
- Other products are not as efficient as neonicotinoids
- Others ____________________

(Note: for the online version, respondents would be automatically directed to the added question based on his choice in Question 22.)

23. If neonicotinoid seed treatments were permanently banned in the future, what alternative pest management strategies would you suggest? Please tick all that apply.

- Stop growing oilseed rape and switch to other crops
- Grow a smaller area of oilseed rape
- Grow oilseed rape less often
- Use new oilseed rape varieties
- Use new insecticides if available
- Use currently available insecticides, please specify product names: ____________________
- Do nothing
- No opinions
- Others, please state: ____________________

24. After the neonicotinoid restriction, what are the expected yields for winter oilseed rape production for 2014/15 (compare with typical yields in the last 5 years)?

- < 3 t/ ha less
- 2-3 t/ ha less
- 1-2 t/ ha less
- 0-1 t/ ha less
- about the same
- 0-1 t/ ha more
- 1-2 t/ ha more
- 2-3 t/ ha more
- >3 t/ ha more
- not sure

25. After the neonicotinoid restriction, what are the expected yields for spring oilseed rape production for 2014/15 (compare with typical yields in the last 5 years)?

- < 3 t/ ha less
- 2-3 t/ ha less
- 1-2 t/ ha less
- 0-1 t/ ha less
- about the same
- 0-1 t/ ha more
- 1-2 t/ ha more
- 2-3 t/ ha more
- >3 t/ ha more
- not sure

26. Following the restriction, did oilseed rape farmers spend more money on insecticide products in 2014/15 (compare with 2013/14)?

- Yes, much more
- Yes, but just a bit more
- About the same
- No, a bit less
- No, much less
- Not sure

27. Following the restriction, did you and/ or other agronomists spend more time to inspect the oilseed rape fields for arthropod pests’ abundance in 2014/15 (compare with 2013/14)?

- Yes
- No
- Not sure

28. Do general seed treatments and sprays have negative influences on the following aspects? Please use the 0-5 influence levels (0 being no influence, 5 being greatest influence).

|  | Users’ health | Natural enemies | Pollinators | Water | Soil |
| --- | --- | --- | --- | --- | --- |
| Seed treatments |  |  |  |  |  |
| Sprays |  |  |  |  |  |

Section 5: Feedback

1. Do you have any comments/ suggestions about the contents and structure of the questionnaire? Thank you very much!
