# Supplementary material for: Arthropod Pest Control for UK Oilseed Rape – Comparing Insecticide Efficacies, Side Effects and Alternatives
Source: PLoS One. 2017 Jan 11;12(1):e0169475. doi: 10.1371/journal.pone.0169475 (PMC5226783; doi:10.1371/journal.pone.0169475)
Supplement: S3 Appendix — (DOCX) [file pone.0169475.s003.docx]

**S3 Appendix. Regional distributions**

Methods

In order to take into account regional differences in pest damage and controls for oilseed rape, respondents were asked to mention the regions they referred to at the beginning of survey. Defra classification of UK regions was used. If a respondent represented several cities or counties, these were re-categorised into regions. If a respondent represented several regions (e.g., South East and South West), we assumed that his/ her opinion on each individual region was the same and recorded the same answers for both regions. If a respondent had not mentioned any regions (left blank), we assumed they were for UK in general. Because of the large range covered, if a respondent answered ‘England’ or ‘UK’, they were used as two separate regions.

Results (Table C.1)

Most people represented east (20% out of 90) or south east (~19%) areas when providing information for oilseed rape and neonicotinoid sections, where most oilseed rape is grown in the UK. About 18% represented the midlands, and 5% north. Six respondents were from Scotland, two from Northern Ireland and one from Wales. Ten respondents have not had specific regions, so we assume they represented UK in general (in total 22).

**Table C.1**

Regional distributions of respondents for oilseed rape and neonicotinoid sections

| Regions | One | Two | Three | Four | Total |
| --- | --- | --- | --- | --- | --- |
| East | 16 | 1 | 1 |  | 18 |
| South East | 10 | 5 | 1 | 1 | 17 |
| South West | 2 | 4 | 2 | 1 | 9 |
| East Midlands | 4 | 1 | 1 | 1 | 7 |
| West Midlands | 6 | 1 | 1 | 1 | 9 |
| Yorkshire & the Humber | 6 |  |  |  | 6 |
| North East | 2 | 1 |  |  | 3 |
| North West |  | 2 |  |  | 2 |
| Scotland | 6 |  |  |  | 6 |
| Northern Ireland | 2 |  |  |  | 2 |
| Wales | 0 | 1 |  |  | 1 |
| England | 4 |  |  |  | 4 |
| UK | 22 |  |  |  | 22 |

Note: ‘1’ is the counts for respondents who only mentioned one region; ‘2’ is for those who mentioned two regions, and so on.
