# Supplementary material for: Arthropod Pest Control for UK Oilseed Rape – Comparing Insecticide Efficacies, Side Effects and Alternatives
Source: PLoS One. 2017 Jan 11;12(1):e0169475. doi: 10.1371/journal.pone.0169475 (PMC5226783; doi:10.1371/journal.pone.0169475)
Supplement: S4 Appendix — (DOCX) [file pone.0169475.s004.docx]

**S4 Appendix. Hazard Quotient approach**

The Hazard Quotient approach for the non-target arthropod species (*HQ_N_*) has been developed by the ‘European Standard Characteristics of Non-target Arthropod Regulatory Testing’ workshop [1], and has been used as a required assessment of pesticide registration in the EU. The in-field *HQ_N_* method is:

$$In-field {HQ}_{N}=\frac{Application rate\times MAF}{{LR}_{50}(N)}$$

where the ‘application rate’ is the single application rate in g a.i. (active ingredient) /ha, MAF is the Multiple Application Factor (maximum residue level after multiple applications of the products), *LR*_50_(*N*) is the lethal rate 50 for natural enemies in g a.i. /ha (application rate causing 50% mortality of the organisms under worst-case laboratory conditions). This method is used to assess the initial toxicity experiment of the two most sensitive non-target arthropod species, *Aphidius rhopalosiphi* and *Typhlodromus pyri*. If one or both the HQ results exceed 2, it indicates a potential hazard to the non-target species. In this study due to lack of information on the toxicity test for *Typhlodromus pyri*, only *Aphidius rhopalosiphi* is used.

The application rates of the available active ingredients were estimated from the Fera pesticide use database [2]. It is assumed that the derived application rate is the single application rate. As for the MAF, because the average spray rounds of insecticides used in the UK in the past 14 years is 1.1 (calculated from [2]), it is assumed that MAF is 1 for all the active ingredients.

*LR*_50_(*N*) were mainly collected from the European Food Safety Authority [3]:

| Active ingredients | Chemical groups | *LR*_50_(*N*) (g/ha) |
| --- | --- | --- |
| Pirimicarb | Carbamate | 620 |
| Lambda-cyhalothrin | Pyrethroid | 0.59 |
| Dimethoate | Organophosphate | 0.014 |
| Thiacloprid | Neonicotinoid | 6.8 |
| Indoxacarb | Oxadiazine | 49 |
| Pymetrozine | Azomethine | 75 |

**References**

1. ESCORT. Guidance document on regulatory testing and risk assessment procedures for plant protection products with non-target arthropods. European Standard Characteristics of Non-Target Arthropod Regulatory Testing, Wageningen, the Netherlands. 2000.

2. Fera. Pesticide Usage Surveys; 2015. Fera Science Ltd [Internet]. Accessed: http://pusstats.fera.defra.gov.uk/index.cfm.

3. EFSA. Rapporteur Member State assessment reports submitted for the EU peer review of active substances used in plant protection products. European Food Safety Authority. 2015. Available: http://dar.efsa.europa.eu/dar-web/provision.
