# Supplementary material for: Arthropod Pest Control for UK Oilseed Rape – Comparing Insecticide Efficacies, Side Effects and Alternatives
Source: PLoS One. 2017 Jan 11;12(1):e0169475. doi: 10.1371/journal.pone.0169475 (PMC5226783; doi:10.1371/journal.pone.0169475)
Supplement: S5 Appendix — (DOCX) [file pone.0169475.s005.docx]

**S5 Appendix. Other figures and tables**

Fig. E.1. Organization distribution for respondents to the neonicotinoid section. Figures on the pie charts are the number of respondents. In total 90 respondents provided this information (N=90).

**Table E.1**

Weighted average response for the three main arthropod pests in UK oilseed rape by regions. Numbers in brackets are the number of respondents.

|  | Aphids | Peach—potato aphid | Cabbage aphid | Cabbage stem weevil | Brassica pod midge | Beetles | Pollen beetle | Cabbage stem flea beetle | Weevils | Cabbage seed weevil |
| --- | --- | --- | --- | --- | --- | --- | --- | --- | --- | --- |
| East (18) | 1.17 | 1.33 | 0.33 | 0.00 | 0.50 | 0.50 | 3.83 | 6.67 | 1.33 | 0.33 |
| South East (17) | 1.83 | 1.67 | 0.17 | 0.00 | 0.67 | 0.00 | 3.83 | 6.67 | 0.67 | 0.17 |
| South West (9) | 0.50 | 1.50 | 0.17 | 0.17 | 0.33 | 0.00 | 1.83 | 3.67 | 0.67 | 0.00 |
| East Midlands (7) | 0.00 | 1.50 | 0.17 | 0.00 | 0.00 | 0.00 | 1.00 | 1.83 | 0.50 | 0.33 |
| West Midlands (9) | 0.67 | 1.33 | 0.17 | 0.00 | 0.67 | 0.00 | 1.67 | 2.67 | 0.33 | 0.00 |
| York (6) | 0.00 | 0.17 | 0.00 | 0.00 | 1.00 | 0.00 | 1.00 | 1.83 | 0.00 | 0.67 |
| North East (3) | 0.00 | 0.00 | 0.00 | 0.00 | 0.33 | 0.00 | 0.83 | 1.33 | 0.17 | 0.33 |
| North West (2) | 0.17 | 0.00 | 0.00 | 0.00 | 0.17 | 0.00 | 0.00 | 0.83 | 0.00 | 0.33 |
| Scotland (6) | 0.33 | 0.67 | 0.00 | 0.83 | 0.00 | 0.50 | 0.83 | 1.33 | 0.33 | 0.17 |
| Northern Ireland (2) | 0.50 | 0.00 | 0.33 | 0.00 | 0.00 | 0.00 | 0.50 | 0.33 | 0.00 | 0.17 |
| Wales (1) | 0.17 | 0.00 | 0.00 | 0.00 | 0.00 | 0.00 | 0.00 | 0.33 | 0.00 | 0.00 |
| England (4) | 0.00 | 0.00 | 0.50 | 0.00 | 0.17 | 0.00 | 0.83 | 1.83 | 0.33 | 0.17 |
| UK (19) | 1.00 | 2.50 | 0.67 | 0.17 | 0.67 | 1.67 | 3.50 | 4.67 | 0.83 | 1.00 |

Fig. E.2. Percentage of respondents’ opinions on the restriction on the neonicotinoid seed treatments in oilseed rape (Total number of respondents = 90).

**Table E.2**

Pairwise comparisons among organizations of the opinions on the neonicotinoid seed treatments restriction in UK oilseed rape. Test method is Fisher’s exact test (with Monte Carlo simulated p value, 100,000 replicates), level of significance is 0.05.

1. *oppose* (including ‘Oppose’ and ‘Strongly oppose’) versus *favour* (including ‘Favour’ and ‘Strongly favour’)

|  | Universities | Private research institutes | Agri-chemical companies | Commercial consulting firms | Growers | Independent consultants |
| --- | --- | --- | --- | --- | --- | --- |
| Government | 1 | 1 | 0.6 | 1 | 0.6 | 0.04 |
| Universities |  | 1 | 0.4 | 0.6 | 0.2 | 0.007 |
| Private research institutes |  |  | 0.6 | 0.5 | 0.5 | 0.08 |
| Agri-chemical companies |  |  |  | 1 | 1 | 0.2 |
| Commercial consulting firms |  |  |  |  | 1 | 0.3 |
| Growers |  |  |  |  |  | 0.4 |

1. having an opinion (*oppose*/ *favour*) versus ‘Neutral’/ ‘Not sure’

|  | Universities | Private research institutes | Agri-chemical companies | Commercial consulting firms | Growers | Independent consultants |
| --- | --- | --- | --- | --- | --- | --- |
| Government | 0.7 | 1 | 0.1 | 0.3 | 0.1 | 0.05 |
| Universities |  | 1 | 0.02 | 0.2 | 0.03 | 0.005 |
| Private research institutes |  |  | 0.07 | 0.3 | 0.09 | 0.06 |
| Agri-chemical companies |  |  |  | 1 | 1 | 1 |
| Commercial consulting firms |  |  |  |  | 1 | 1 |
| Growers |  |  |  |  |  | 1 |

**Table E.3**

Pairwise comparisons among organizations of whether they have provided farming advice services from 2009/10 to 2013/14. Test method is Fisher’s exact test (with Monte Carlo simulated p value, 100,000 replicates), level of significance is 0.05.

|  | Universities | Private research institutes | Agri-chemical companies | Commercial consulting firms | Growers | Independent consultants |
| --- | --- | --- | --- | --- | --- | --- |
| Government | 0.05 | 0.3 | 0.7 | 1 | 0.2 | 0.05 |
| Universities |  | 0.7 | 0.02 | 0.08 | 0.0009 | 0.000001 |
| Private research institutes |  |  | 0.1 | 0.3 | 0.009 | 0.0008 |
| Agri-chemical companies |  |  |  | 1 | 0.3 | 0.2 |
| Commercial consulting firms |  |  |  |  | 0.5 | 0.2 |
| Growers |  |  |  |  |  | 1 |

**Table E.4**

First reported year for insecticide resistance in main arthropod pests in UK oilseed rape

| Species | Resistance target sites | Affected chemical groups | Approximate Resistance first reported date |
| --- | --- | --- | --- |
| Pollen beetle | kdr | Pyrethroids | 2006 [1] |
| Cabbage stem flea beetle | kdr | Pyrethroids | 2014 [2] |
| Peach–potato aphid | kdr | Pyrethroids | 1997 [3] |
|  | E4 | Mainly OPs, also Carbamates and Pyrethroids | Early 1970s [3] |
|  | MACE | Carbamates | 1996 [3] |

**References:**

1. HGCA. Controlling pollen beetle and combating insecticide resistance in oilseed rape. Home Grown Cereals Authority, Kenilworth,UK. 2011.

2. IRAC. Cabbage stem flea beetle (CSFB), Psylliodes chrysocephala-Resistance on the move. Insecticide Resistance Action Committee. 2015.

3. IRAG. Guidelines for preventing and managing insecticide resistance in the peach-potato aphid , Myzus persicae. Insecticide Resistance Action Group. 2005.
